# Identification and Functional Characterization of FLOWERING LOCUS T in *Platycodon grandiflorus*

**DOI:** 10.3390/plants11030325

**Published:** 2022-01-26

**Authors:** Gayeon Kim, Yeonggil Rim, Hyunwoo Cho, Tae Kyung Hyun

**Affiliations:** 1 Department of Industrial Plant Science and Technology, Chungbuk National University, Cheongju 28644, Korea; gayeon0508@gmail.com; 2 Plant Molecular Biology and Biotechnology Research Center, Gyeongsang National University, Jinju 52828, Korea; ssac1@gnu.ac.kr

**Keywords:** CONSTANS, FLOWERING LOCUS T, *Platycodon grandiflorus*, temperature

## Abstract

*Platycodon grandiflorus* roots have been used as a foodstuff and traditional medicine for thousands of years in East Asia. In order to increase the root development of *P*. *grandiflorus*, cultivators removed the inflorescences, suggesting the possible negative effect of flowering on root development. This indicates that the genetic improvement of *P*. *grandiflorus* by late flowering is a potential approach to increase productivity. However, nothing is known about key genes integrating multiple flowering pathways in *P*. *grandiflorus*. In order to fill this gap, we identified potential homologs of the *FLOWERING LOCUS T* (*FT*) gene in *P*. *grandiflorus*. The alignment with other FT members and phylogenetic analysis revealed that the *P*. *grandiflorus* FT (PlgFT) protein contains highly conserved functional domains and belongs to the FT-like clade. The expression analysis revealed spatial variations in the transcription of *PlgFT* in different organs. In addition, the expression level of *PlgFT* was increased by high temperature but not by photoperiodic light input signals, presumably due to lacking the CONSTANS binding motif in its promoter region. Furthermore, PlgFT induced early flowering upon its overexpression in *P*. *grandiflorus*, suggesting the functional role of PlgFT in flowering. Taken together, we functionally characterized PlgFT as a master regulator of *P*. *grandiflorus* flowering under inductive high temperature, which will serve as an important target gene for improving the root productivity.

## 1. Introduction

The transition from vegetative to the reproductive stage is a critical event in plant development. The floral transition is controlled by diverse endogenous and environmental cues such as plant age, autonomous pathway, gibberellic acid (GA), photoperiod, vernalization, and ambient temperature change [1,2]. In Arabidopsis, the master regulator of floral transition, FLOWERING LOCUS T (FT) protein, is synthesized in the phloem companion cell of the leaf, translocated into a sieve tube system, and moves to shoot apical meristem (SAM) to systematically reprogram the floral gene network under the inductive photoperiod [3,4]. At the Arabidopsis SAM, FT physically interacts with basic leucine-zipper (bZIP)-type transcription factor FLOWERING LOCUS D (FD) and 14-3-3 proteins. These interactors form florigen activation complex (FAC) in Arabidopsis to promote the floral meristem identity genes such as *APETALA1* (*AP1*) [5] and *SUPPRESSOR OF OVEREXPRESSION OF CONSTANS1* (*SOC1*) [6] either directly or indirectly. In addition, CONSTANS (CO) functions as the master regulator of photoperiodic induction of FT expression under long-day (LD) and short-day (SD) conditions [3,7,8,9], whereas the flowering of day-neutral plants is not affected by the CO-FT module [3,10,11]. Currently, ambient temperature-responsive mechanisms have also been reported as regulating FT-mediated flower induction [12]. In Arabidopsis, PHYTOCHROME INTERACTING FACTOR4 (PIF4) directly binds the *FT* promoter to activate *FT* at high temperature, resulting in the early flowering under short-day conditions [13]. In addition, the Arabidopsis FT homolog FT-like (*Narcissus FLOWERING LOCUS T1*) *NFT1* gene was up-regulated by heat treatment (30 °C) and promoted flower initiation [14]. These findings indicate the importance of ambient temperature to induce flowering.

*Platycodon grandiflorus*, a day-neutral and herbaceous perennial plant, is mainly cultivated for its storage roots. The roots of *P*. *grandiflorus* were used as traditional herbal medicine and functional foodstuff in East Asia due to their considerable bioactive components, such as triterpenoid saponin [15]. In *P*. *grandiflorus*, the transition from vegetative to reproductive growth has been known to have an inhibitory effect on storage root development, presumably due to the consumption of accumulated energy source (starch) from their storage root and delayed energy distribution to the bidirectional source to sink flow for flowering [16,17]. This indicates that delayed flowering time should affect root development via the availability of more time for vegetative growth. It was shown that the flowering timing of *P*. *grandiflorus* was not affected by photoperiod and gibberellin together but was significantly accelerated by high temperature [18]. Similarly, the day-natural tomato flowering is regulated by the FT-homologue *SFT/SP3D* gene, which is regulated independently of CO and day length [11,19]. In plants, the flowering transition and inflorescence architecture are modulated by eukaryotic phosphatidylethanolamine-binding protein family, which contains TWIN SISTER OF FT (TSF), TERMINAL FLOWER 1 (TFL1), ARABIDOPSIS THALIANA CENTRORADIALIS (ATC), BROTHER OF FT AND TFL1 (BFT), and MOTHER OF FT AND TFL1 (MFT) [20]. Among them, FT functions as the mobile flower-promoting signal, which promotes the transition to reproductive development and flowering, whereas TFL1 plays as a repressor of this transition [21]. Although MFT also functions as a floral inducer [22], the core determinant gene for floral transition is assumed to be FT in higher plants [23]. However, in *P*. *grandiflorus*, the information of multiple flowering pathways is completely unknown due to the missing genetic information about floral inducers such as FT.

Therefore, in this study, we identified *P*. *grandiflorus FT* (*PlgFT*) and analyzed the expression pattern of *PlgFT* in response to gibberellin and high temperature (30 °C) to investigate its role in the flowering mechanism of *P*. *grandiflorus*. Overexpression of *PlgFT* resulted in the induction of early flowering in *P*. *grandiflorus*, which suggests that *PlgFT* is potentially a gene resource for improving storage root development using the plant molecular breeding approach in this day-neutral, economically important medicinal plant.

## 2. Results and Discussion

### 2.1. Identification of FT Gene in P. grandiflorus

**Figure 1:**
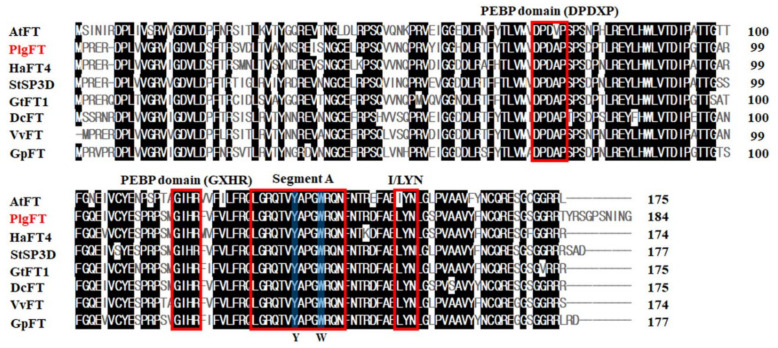
Sequence alignment of PlgFT protein and other reported FT proteins. Red boxes indicate the conserved motifs and segments, including DPDxP, GXHR, segment A, and LYN. Blue boxes indicate key amino acids for flowering activator.

FT is well known as a master flowering regulator, and the amino acid sequences are highly conserved in many flowering plants [23]. In order to investigate the *FT-like* gene integrating multiple flowering pathways in *P*. *grandiflorus*, an *FT-like* gene was identified in the *P*. *grandiflorus* genome data [24] using the sequences of FTs from various species and was cloned for functional characterization. The *PlgFT* gene was found to have an open reading frame of 555 bp with a stop codon, encoding a protein of 184 amino acids (Figure 1). The PlgFT protein conserved the PEBP domain (DPDxP and GxHR) and key amino acid residues (Gln139 (Q139) and Tyr84 (Y84)), similar to Arabidopsis FT [25]. In addition, segment A (LGRQTVYAPGWRQN), which forms an external loop and is required for FT proteins to act as a floral activator [26], and LYN, which is required for FT full functionality with segment A [25], were found in PlgFT protein. In flowering pathways, FT and TFL1 act as flowering regulators with homology to phosphatidylethanolamine-binding proteins [20,21]. Although *Arabidopsis* FT exhibits protein sequence similarity of ~60% with *Arabidopsis* TFL1, they function in an opposite manner [21,27]. Based on the sequence alignment of FT-like proteins, flowering inducer and repressor functions in FT-like proteins were distinguished by two conserved sites at positions 134 and 138 (based on Arabidopsis FT protein sequence) [21]. As shown in Figure 1, PlgFT protein contains tyrosine at position 134 and tryptophan at position 138, similar to all flowering inducer FTs, indicating that putative PlgFT protein belongs to the flowering inducer FT.

In order to explore the phylogenic relationships between the FT-like proteins from various species, a phylogenetic tree was constructed using the neighbor-joining method. As shown in Figure S1, PlgFT protein belongs to the FT clade but is not grouped into TFL clades. In addition, PlgFT is located genetically close to GtFT and StSP3D, which are well-known flowering inducers [28,29], collectively suggesting that PlgFT could be a functional ortholog of FT, which acts as a flowering inducer.

**Figure 2:**
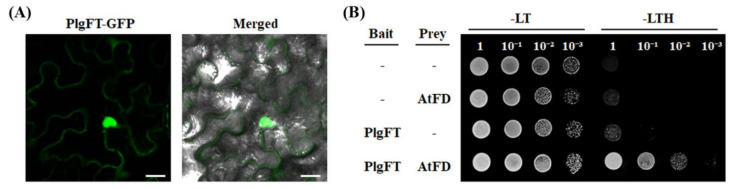
Subcellular localization and yeast two-hybrid assay for *P*. *grandiflorus* FT. (**A**) Subcellular localization of PlgFT in *Nicotiana benthamiana* leaves. PlgFT recombinant proteins transiently expressed in *N*. *benthamiana* leaves through agro-infiltration. Scale bar = 20 μm. (**B**) Analysis of protein–protein interaction between PlgFT and Arabidopsis FD (AtAD) using yeast two-hybrid assay. Yeast cells (AH109) were grown on SD agar medium lacking either Trp and Leu (−LT) or Trp, Leu, and His (−LTH).

The analysis of protein subcellular localization is important for further understanding its function. In order to investigate the subcellular localization of PlgFT in plants, we tested a transient expression system using an Agro-infiltration approach. The green fluorescence protein (GFP) fused to PlgFT was expressed under the control of the 35S promoter. As shown in Figure 2A, PlgFT-GFP was localized in both the nucleus and cytoplasm. This result was similar to the subcellular localization pattern of previously studied FT proteins in various plants, including switchgrass, sweet cherry, alfalfa, carrot, tobacco, and *Eriobotrya deflexa* [30,31,32,33,34,35]. 

FT and FD are known to be interdependent partners in protein interaction for their transcriptional regulation, then FT-FD complex binds and promotes the expression of floral identity genes in SAM [5]. Therefore, we performed a yeast two-hybrid assay to test whether PlgFT can interact with FD. Arabidopsis FD (AtFD) and PlgFT were used as prey and bait, respectively. As shown in Figure 2B, the transformed yeast expressing PlgFT and AtFD grew in SD medium without Leu, Trp, and His, indicating that PlgFT directly interacts with AtFD. 

### 2.2. Expression Pattern of PlgFT in Various Organs

**Figure 3:**
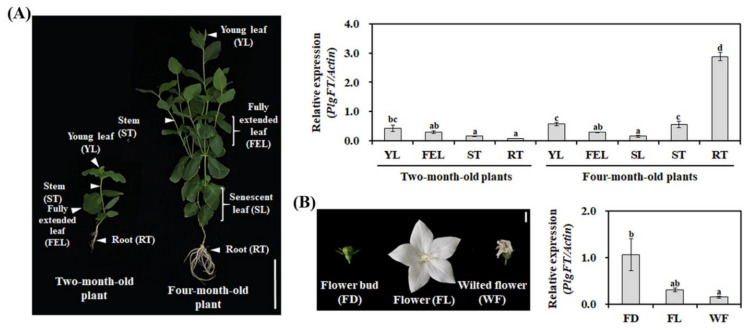
Expression pattern of *PlgFT* in different organs (**A**) and flower development stages (**B**). Expression levels of *PlgFT* were normalized to actin. Data represent the means ± SD of three independent experiments. Different letters correspond to means that are statistically different (*p* < 0.05). The scale bars represent 10 cm (**A**) and 1 cm (**B**).

The analysis of organ-specific transcription can be used to determine the potential role of a gene of interest. Although the expression of Arabidopsis *FT* was detected at the vascular tissue (phloem) of cotyledons and leaves [36], the transcript levels of *FTs* identified from various plants were detected in various tissues, including leaf, flower, fruit, and root [33,37,38]. Therefore, to evaluate the expression profile of *PlgFT*, the transcripts of *PlgFT* were monitored by RT-qPCR in various organs of *P*. *grandiflorus,* including the leaves, stems, flowers, and roots. As shown in Figure 3A, *PlgFT* was highly expressed in unexpanded young leaves compared to fully expanded leaves and senescent leaves. In four-month-old plants, *PlgFT* was highly expressed in the root, followed by unexpanded young leaves and stems. In onion (*Allium cepa*), *FT* genes are not only involved in flowering but also in the regulation of onion bulb formation [26,39]. In addition, flowering and tuberization are controlled by StSP6A (FT ortholog of potato) in potato [29], indicating that FT also acts as a key mobile signal controlling different organ developments. Therefore, based on the expression pattern of *PlgFT*, it can be hypothesized that PlgFT has a distinct role in storage root development, which requires further investigation.

In the flower development stages, *PlgFT* was highly expressed in the flower bud compared with the flower and wilted flower. In addition, *Eriobotrya deflexa FT* [33], *Jatropha curcas FT* [40], and grapevine *FT* [41] are primarily expressed in the reproductive organs rather than leaves, similar to *PlgFT*. Taken together, the expression pattern of *PlgFT* indicates that PlgFT might play a role in both flower and storage root.

### 2.3. The Diurnal Expression Pattern of PlgFT

**Figure 4:**
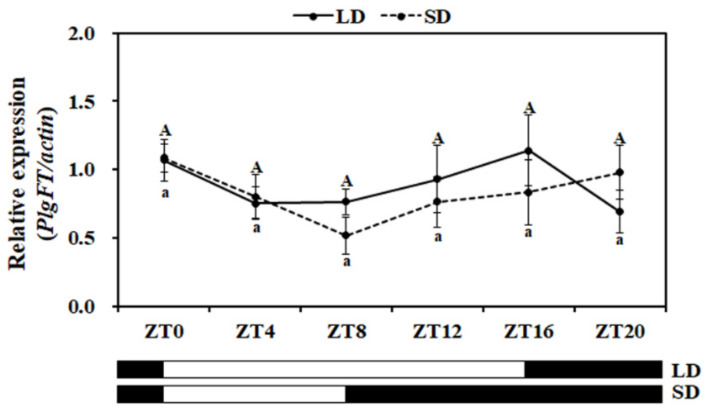
Diurnal expression pattern of *PlgFT* under different photoperiodic conditions. The expression levels of *PlgFT* were measured every 4 h throughout the diurnal cycle in LD and SD conditions. Expression levels of *PlgFT* were normalized to actin. Values represent three independent replicates ± SD. Uppercase denotes significance (*p* < 0.05) between the expression levels under LD, whereas lowercase denotes significance (*p* < 0.05) between the expression levels under SD.

During photoperiodic flowering, CO integrates multiple upstream signals to respond to the photoperiod for the optimal flowering time. In both *Arabidopsis* and rice, the expression of *CO* and *CO* ortholog *Hd1* have distinct diurnal expression profiles [3,8,9], indicating that expression of *FT* is controlled by the circadian clock. In order to investigate the diurnal expression pattern of *PlgFT*, the transcription level of *PlgFT* was investigated over a 24 h period. *PlgFT* exhibited similar expression levels throughout the LD or SD-diurnal time course (Figure 4), indicating that *PlgFT* is not diurnally regulated. 

The circadian clock regulation of *CO* transcription is crucial for the expression of *FT* [4]. In the *Arabidopsis FT* promoter region, two CO-responsive elements (CORE1 and CORE1) with consensus sequence TGTG(N2-3)ATG were identified [42]. The mutation in the CORE1 reduced the expression of *FT* and resulted in later flowering. In addition, the late-flowering phenotype of *ft-10* plants was not recovered by *FT* under the control of the native promoter lacking the COREs [7], which indicates that CO-mediated *FT* expression is mediated by COREs. However, as shown in Figure S2, the *PlgFT* promoter region did not contain a consensus sequence corresponding to COREs [7]. Taken together, the absence of the CO binding motif in the promoter region led to the non-responsiveness of *PlgFT* expression under light conditions.

### 2.4. Effects of High Temperature and GA3 on PlgFT Expression

**Figure 5:**
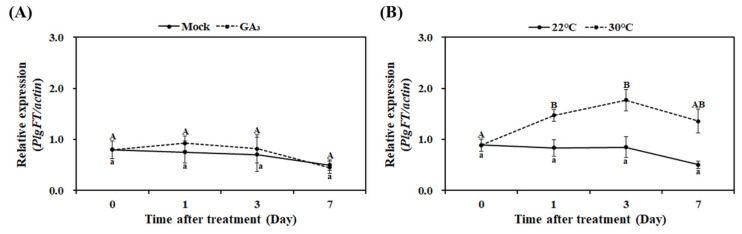
Regulation of *PlgFT* expression by GA_3_ (**A**) and high-temperature conditions (**B**). Two-month-old plants were treated with GA_3_ or high temperature (30 °C). The expression levels of *PlgFT* were normalized to actin. Values represent three independent replicates ± SD. Uppercase denotes significance (*p* < 0.05) between the expression levels after treatment of GA3 or high temperature (30 °C), whereas lowercase denotes significance (*p* < 0.05) between the expression levels after treatment of mock or normal temperature (22 °C).

As described above, the floral transition is controlled not only by the photoperiod but also by endogenous and environmental cues, including ambient temperature change and phytohormone GA. In *P*. *grandiflorus*, flowering is accelerated at elevated temperatures, whereas the treatment of GA_3_ does not affect flowering [18,43], indicating the importance of ambient temperature to *P*. *grandiflorus* flowering. In order to further support the effect of high temperature on *P*. *grandiflorus* flowering, variation in *PlgFT* expression was determined after the treatment with high temperature or GA_3_. As shown in Figure 5, the GA_3_ application did not change the expression level of *PlgFT*, whereas the high-temperature treatment (30 °C) strongly induced *PlgFT* expression (2.5-fold) compared to the control plant (22 °C). The increment of *PlgFT* expression continued for seven days by high-temperature treatment, indicating that the high-temperature flowering response is mediated by the induction of *PlgFT* in *P*. *grandiflorus*. In higher plants, the levels of *FT* transcription correlated with temperature effects on floral initiation. The expression of *FaFT3* (*Fragaria* × *ananassa FT3*) increased by low temperature, in accordance with the result that these treatments promoted floral initiation, whereas high temperature inhibited the expression of *FaFT3* and floral initiation [44]. However, contrary to vernalization, high temperature provided a flowering signal by increasing *NtFT* (Narcissus *FT* gene homolog) expression, whereas the down-regulation of *NtFT* by low temperature inhibited flower induction [45]. In addition, high-temperature promoted soybean flowering through the induction of soybean floral activators (soybean *FT* homologs *GmFT2a* and *GmFT5a*) and the suppression of floral repressors [46]. Under high-temperature conditions, the histone variant H2A.Z plays a pivotal role in temperature-dependent *FT* transcription by PHYTOCHROME INTERACTING FACTOR 4 (PIF4) and key thermosensory bHLH transcription factor by improving the accessibility of PIF4 to the *FT* promoter [4]. Our data revealed that high-temperature functions as an activator of *PlgFT* expression, but further studies are remained to identify and characterize the high temperature-mediated H2A.Z-PIF4 regulatory module in *P*. *grandiflorus*.

### 2.5. Ectopic Overexpression of PlgFT in P. grandiflorus Accelerates flowering

**Figure 6:**
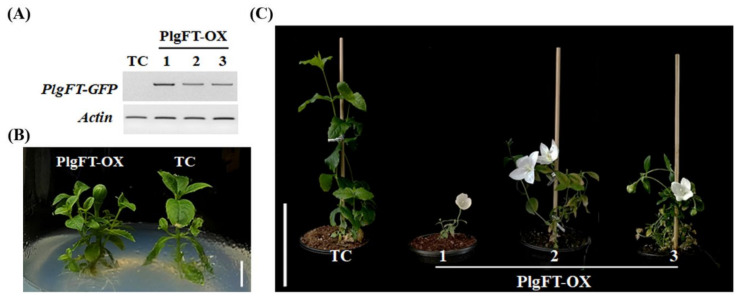
Ectopic expression of *PlgFT* in *P*. *grandiflorus* induced early flowering. (**A**) The expression of *PlgFT-GFP* in the selected transgenic plants was confirmed by RT-PCR. Phenotypes of TC and *PlgFT-GFP* overexpression plants (PlgFT-OX) grown in the in vitro condition (**B**) and soil (**C**). Scale bar = 1 cm (**B**) or 10 cm (**C**).

In order to explore the biological function of PlgFT on flowering in *P*. *grandiflorus*, we generated transgenic *P*. *grandiflorus* overexpressing *PlgFT* under the control of the 35S promoter. We obtained several independent transgenic lines and checked the presence of *PlgFT*-*GFP* transgene by reverse transcription (RT)-PCR (Figure 6A). In addition, we selected regenerated plants that survived on hygromycin selection medium but showed no transcription level of *PlgFT*-*GFP* as transgenic controls (TCs). Overexpression of *PlgFT*-*GFP* induced extreme early flowering compared to the TC in both the medium and soil growth condition (Figure 6B,C). In addition, *PlgFT*-*GFP* overexpressing plants exhibited dwarfism and the early leaf senescence phenotype; this should be mediated by the consequence of growth deficiency due to the short vegetative growth period and premature transition to the reproductive phase. In storage root plants, including cassava, photoassimilate translocation from leaves to roots is very important for the bulking of storage roots [47]. This indicates that the short vegetative growth period by *PlgFT*-*GFP* overexpression should affect the bulking of storage roots. Unlike *PlgFT*-*GFP* overexpressing plants, the overexpression of *StSP6A* induces the early flowering and tuberization without early leaf senescence phenotype [29]. Therefore, the inducible promoter would be required for analyzing the function of PlgFT in the development of storage roots. Based on the extreme early flowering phenotype caused by the ectopic expression of *PlgFT*-*GFP*, we conclude that PlgFT is a functional ortholog of Arabidopsis FT and acts as a flowering-inducer in *P*. *grandiflorus*.

## 3. Materials and Methods

### 3.1. Plant Materials and Experimental Treatment

*P*. *grandiflorus* were sown and grown in soil under long-day condition (LD, 16 h light/8 h dark) or short-day condition (SD, 8 h light/16 h dark) at 22 °C. In order to analyze the organ-specific expression pattern of *PlgFT*, various organs of *P*. *grandiflorus,* including the leaves, stems, and roots, were harvested from two- or four-month-old plants, which were grown under LD. The bud, flowers, and wilted flowers were harvested from four- to five-month-old plants. Plants entered into the adult vegetative phase were used for analyzing the diurnal expression pattern of *FTs* [48,49]. Thus, the diurnal expression patterns of *PlgFT* were investigated over a 24 h period using three-month-old plants. For GA_3_ treatment, GA_3_ solution was prepared using 1% EtOH and 0.02% tween-20. Three-month-old plants (LD condition) were sprayed with 1 mM GA_3_ or with a solution containing 1% EtOH and 0.02% tween-20 as a mock control. In addition, plants were transferred to a growth chamber (30 °C, LD conditions) to investigate the effect of temperature. The unexpanded young leaves were collected at different time points after treatment (0 d, 1 d, 3 d, and 7 d), immediately frozen in liquid nitrogen, and stored at −80 °C prior to analysis.

### 3.2. Identification FT Gene in P. grandiflorus

In order to identify FT in *P*. *grandiflorus*, the *P*. *grandiflorus* genomic sequence data [24] were searched using FT protein sequences obtained from different plant species as direct queries with BLAT-search. The protein sequence of putative PlgFT was aligned with FT and TFL proteins in different plant species, and a phylogenetic tree using the condensed alignment in MEGA7.0 with the neighbor-joining algorithm was constructed. Table S1 lists the accession number of proteins used for the analyses of multiple-sequence alignment and phylogenetic tree.

### 3.3. Plasmid Construction

The full-length *PlgFT* gene was amplified using gene-specific primers (Table S2) designed using *P*. *grandiflorus* genomic sequence data. The full-length coding sequence of *PlgFT* was cloned into the pENTR/D-TOPO vector and sub-cloned into a gateway binary vector pGWB505 or pGBKT7 (for yeast two-hybrid analysis). The sequence of *PlgFT* (NS-3080) was deposited in the National Agricultural Biotechnology Information Center (NABIC, http://nabic.rda.go.kr).

### 3.4. RNA Extraction and Quantitative Real-Time PCR

Total RNA was extracted from *P*. *grandiflorus* samples using the FavorPrep Plant Total RNA Purification Mini Kit (Favogen, Pingtung, Taiwan), and 500 ng of total RNA were reverse-transcribed using a cDNA synthesis kit (TOYOBO Co., Ltd., Osaka, Japan). In order to determine the expression levels of the *PlgFT* gene, RT-qPCR was performed using the CFX96TM Real-time system (Bio-Rad, Hercules, CA, USA). The expression levels of *PlgFT* were normalized to the constitutive expression level of *actin* using the 2^−∆∆CT^ method. Table S2 lists the primer sequences.

### 3.5. Subcellular Localization of PlgFT in Tobacco Leaves

For transient *PlgFT*-*GFP* expression, a single colony of *Agrobacterium tumefaciens* GV3101 containing *35S:PlgFT-GFP* (PlgFT-GFP) construct was inoculated into 3 mL LB medium with antibiotics at 28 °C. After culturing overnight, the bacteria were harvested and resuspended in MMA (10 mM MES and 10 mM MgCl_2_ containing 200 mM acetosyringone) to an OD600 of 0.3 and used for syringe agro-infiltration into *Nicotiana benthamiana*. GFP fluorescence was visualized 48 h post-infiltration using a confocal laser scanning microscope.

### 3.6. Yeast Two-Hybrid Assay

The pGBKT7-PlgFT and pGADT7-AtFD (Arabidopsis FD) constructs were co-transformed into yeast strain AH109 according to Matchmaker™ GAL4 Two-hybrid System 3 protocol. The transformed yeast cells were selected on SD medium without Leu and Trp, and then protein–protein interactions were confirmed on SD medium without Leu, Trp, and His. The empty pGBKT7 and pGADT7 vectors were used as negative controls.

### 3.7. Agrobacterium Mediated Transformation

*P*. *grandiflorus* seeds were germinated on a half-strength MS medium under LD, and leaves of three-week-old *P*. *grandiflorus* grown in vitro were used for the Agrobacterium-mediated transformation approach. Leaf explants were co-cultivated with the cell suspension of GV3101 containing 35S: PlgFT-GFP (OD600 = 1.0) for three days under dark condition and then placed on the shoot-induced medium (MS medium containing 1 mg/L 6-benzyladenine, 0.5 mg/L 1-naphthaleneacetic acid, 300 mg/L cefotaxime, 30 mg/L hygromycin, 30 g/L sucrose, and 7.5 g/L plant agar). After shoot formation from explant cultures, shoots were transferred to a BA-free medium for the induction of roots. The transgenic lines showing hygromycin resistance were transplanted in soil, and lines with a high level of PlgFT-GFP protein were selected by RT-PCR. In addition, we selected regenerated plants, which were survived on the selection medium but exhibited no expression of *PlgFT*-*GFP*, as transgenic controls.

### 3.8. Statistical Analysis

All experiments were conducted with independent biological replicates and presented as the mean ± standard division. Statistically significant differences were determined using Duncan’s multiple range tests, and the significance was set at *p* < 0.05.

## 4. Conclusions

Here, we first characterized the *FT* gene in *P*. *grandiflorus*, an economically important medicinal plant in East Asia. *PlgFT* contains highly conserved functional motifs and key amino acid residues necessary for FT function as a floral inducer. *PlgFT* was not diurnally regulated because it lacks CORE motifs in its promoter region. In addition, we suggested that the thermoresponsive flowering of *P*. *grandiflorus* is mediated by the induction of *PlgFT*. Furthermore, ectopic expression of *PlgFT* in *P*. *grandiflorus* clearly suggests that PlgFT acts as a flowering inducer. Our results provide an important starting point for future efforts to understand the molecular mechanisms of flowering in *P*. *grandiflorus*, and will be helpful for further studies to improve storage root development by the control of flowering times.

## Data Availability

The data presented in this study are available on request from the corresponding author. The data are not publicly available due to reasons of privacy.

## Supplementary Materials

The following are available online at https://www.mdpi.com/article/10.3390/plants11030325/s1, Figure S1: Phylogenetic tree analysis of the FT/TFL1 proteins in angiosperms, Figure S2: Sequence alignment of conserved FT promoter region, Table S1: The accession number of proteins used for the analyses of multiple-sequence alignment and phylogenetic tree, Table S2: Primer list used for experiments.
