# Supplementary material for: Identification and Functional Characterization of FLOWERING LOCUS T in Platycodon grandiflorus
Source: Plants (Basel). 2022 Jan 26;11(3):325. doi: 10.3390/plants11030325 (PMC8840131; doi:10.3390/plants11030325)
Supplement: Supplementary file 1 [file plants-11-00325-s001.zip › plants-1433381-supplementary.pdf]

Supplementary Materials:

Table S1. The accession number of proteins used for the analyses of multiple-sequence alignment and phylogenetic tree.

Species	Gene	Accession Number
Arabidopsis thaliana	AtFT	BAA77838.1
	AtTFL1	NP_196004.1
Malus domestica	MdTFL1-1,	BAD10961.1
	MdTFL1-2	BAD10967.1
	MdFT1	BAD08340.1
	MdFT2	BAI77728.1
Arabis alpina	AaTFL1	AEH43348.1
Hordeum vulgare	HvFT1	AAZ38709.1
	HvFT2	ABB99414.1
	HvFT3	ABD75336.2
Beta vulgaris subsp. Vulgaris	BvFT	AEI55782.1
Daucus carota	DcFT	KY768910
Narcissus tazetta var. *chinensis*	NtFT	AFS50163.1
Gentia natriflora	GtFT1	BAK40194.1
	GtFT2	BAK40195.1
	GtTFL1	BAK40196.1
Phaseolus vulgaris	PvTFL1y	ABR53775.2
Ficus carica	FcFT1	BAI60052.1
Vitis vinifera	VvTFL1A	ABI99466.1
	VvFT	ABI99465.1
Petunia x hybrida	PhFT	BAV21621.1
Gypsophila paniculata	GpFT	BAK23998.1
Prunus mume	PmFT	BAH82787.1
	PmTFL1	BAJ14521.1
Lactuca sativa	LsFT	BAK14368.1
Populus nigra	PnTFL1	BAD22599.1
	PnFT1	BAD01612.1
	PnFT2	BAD01561.1
Dimocarpus longan	DlFT1	AEZ63949.1
	DlFT2	AEZ63950.1
Zea mays	ZCN8	ABW96231.1
	ZCN2	ABW96225.1
Medicago truncatula	MtFTa1	AEI99551.1
	MtFTb1	AEI99553.1
	MtFTc	AEI99555.1
Allium cepa	AcFT1	AGZ20207.1
	AcFT2	AGZ20208.1
	AcFT4	AGZ20210.1
Citrus sinensis	CiTFL	AAR04683.1
Pisum sativum	PsFTa1	ADZ05699.1
	PsFTa2	ADZ05700.1
	PsFTb1	ADZ05701.1
	PsFTb2	ADZ05702.1
	PsFTc	ADZ05703.1
	PsTFL1a	AAR03725.1
	PsTFL1c	AAQ20811.1
Pyrus spp.	PpTFL1-1	BAD10962.1
	PpTFL1-2	BAD10968.1
	PcTFL1-1	BAD10963.1
	PcTFL1-2	BAD10969.1
Jatropha curcas	JcFT	AHX74040.1
Solanum tuberosum	StTFL1	ABC24691.1
	StSP3D	AAO31792.1
	StSP6A	AAO31794.1
Cucurbita maxima	Cm-FTL1	ABI94605.1
	Cm-FTL2	ABI94606.1
Oryza sativa	Hd3a	BAB61027.1
	RFT1	BAB78479.1
	RCN1	AAD42895.1
Glycine max	GmFT2a	BAJ33491.1
	GmFT4	AJF40162.1
	GmFT5a	BAJ33494.1
Beta vulgaris	BvFT1	ADM92608.1
	BvFT2	ADM92610.1
Saccharum spp.	ScTFL1	AHZ46122.1
	ScFT1	AHZ46121.1
Helianthus annuus	HaFT1	ADF32943.1
	HaFT4	ADO60987.1
Camellia sinensis	CsFT	BAM83573.1
Nicotiana tabacum	NtFT1	AFS17369.1
	NtFT2	AFS17370.1
	NtFT3	AFS17371.1
	NtFT4	AFS17372.1
Brassica oleracea	BoFT	ACH86033.1

Table S2. Primer list used for experiments.

	Source	Gene	Primer name	Sequences (5'-3')	Accession number
For cloning	*Arabidopsis thaliana*	*FD*	AtFD-F	CACCATGTTGTCATCAGCTAAGC	AT4G35900
			AtFD-R	AAATGGAGCTGTGGAAGACCGT	
	Platycodon grandiflorus	*FT*	PlgFT-F	CACCATGCCGAGGGAGAGAGAC	NS-3080*
			PlgFT-R	TCCATTAATGTTAGACGGACC	
For quantitative Real-Time PCR	*Platycodon grandiflorus*	*Actin*	PlgActin -F	CCATACAGTCCCCATTTATGAAG	JF781303
			PlgActin -R	GCTAACTTCTCCTTCATGTCTCTCA	
		FT	PlgFT-F	TGATATCCCGGGAACCACTG	NS-3080*
			PlgFT-R	CCAATTGCCGAAACAACACG	

* National Agricultural Bio-technology Information Center (NABIC, http://nabic.rda.go.kr)

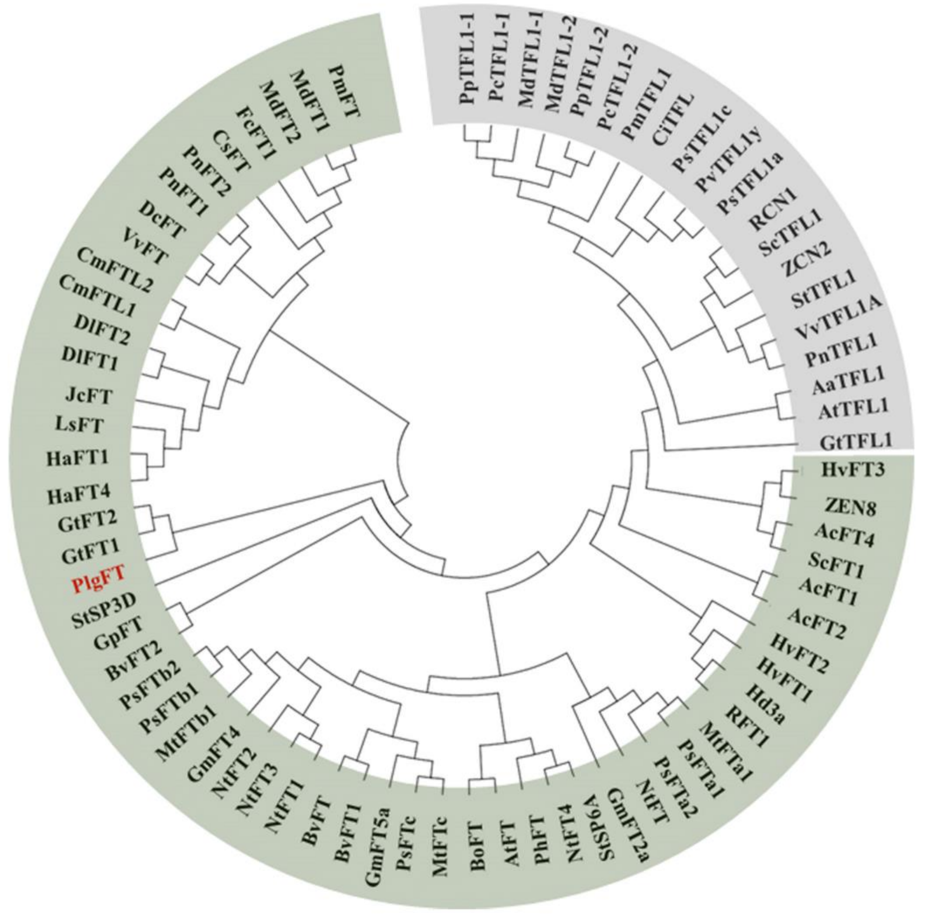

Figure S1. Phylogenetic tree analysis of the FT/TFL1 proteins in angiosperms. FT and TFL1 groups were marked with green and grey color boxes, respectively. A phylogenetic tree was constructed using the Neighbor-joining tree method in MEGA7 software.

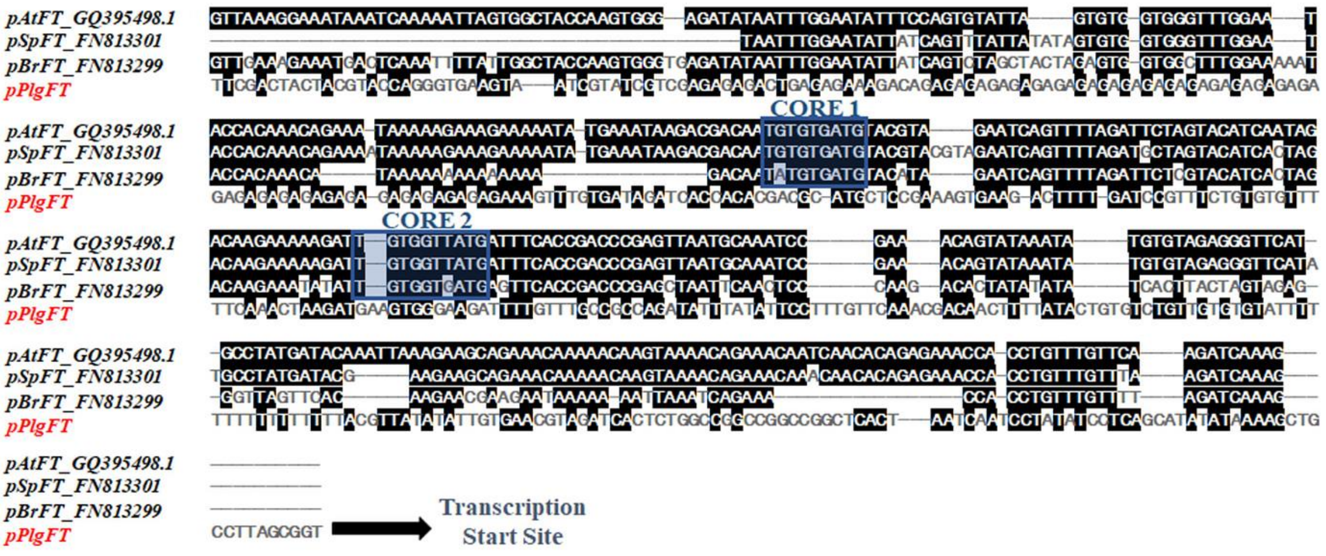

Figure S2. Sequence alignment of conserved *FT* promoter region. Proximal promoter region of *PlgFT* (-1bp to -400bp) aligned block A sequence from other species.
